# Early intervention at home in infants with congenital brain lesion with CareToy revised: a RCT protocol

**DOI:** 10.1186/s12887-018-1264-y

**Published:** 2018-09-05

**Authors:** Giuseppina Sgandurra, Elena Beani, Matteo Giampietri, Riccardo Rizzi, Giovanni Cioni, the CareToy-R Consortium, Francesca Cecchi, Maria Luce Cioni, Carlo Dani, Paolo Dario, Marco Di Galante, Ugo Faraguna, Patrizio Fiorini, Paolo Ghirri, Irene Mannari, Martina Maselli, Valentina Menici, Filomena Paternoster

**Affiliations:** 1 Department of Developmental Neuroscience, IRCCS Fondazione Stella Maris, Viale del Tirreno 331, Calambrone, 56128 Pisa, Italy; 2 Department of Clinical and Experimental Medicine, University of Pisa, Via Roma, 56125 Pisa, Italy; 3 Neonatal Intensive Care Unit, Pisa University Hospital “Santa Chiara”, Via Roma 67, 56126 Pisa, Italy; 4 Neuroscience Center of Excellence and Neonatal Intensive Care Unit, “A. Meyer” University Children’s Hospital, Florence, Italy

**Keywords:** Early intervention, High-risk infants, Randomized clinical trial, Tele-rehabilitation, Information and communication technology, Neurodevelopmental bioengineering, Cerebral palsy, Infant massage

## Abstract

**Background:**

Congenital brain lesions expose infants to be at high-risk for being affected by neurodevelopmental disorders such as cerebral palsy (CP). Early interventions programs can significantly impact and improve their neurodevelopment. Recently, in the framework of the European CareToy (CT) Project (www.caretoy.eu), a new medical device has been created to deliver an early, intensive, customized, intervention program, carried out at home by parents but remotely managed by expert and trained clinicians. Reviewing results of previous studies on preterm infants without congenital brain lesion, the CT platform has been revised and a new system created (CT-R).

This study describes the protocol of a randomised controlled trial (RCT) aimed to evaluate, in a sample of infants at high-risk for CP, the efficacy of CT-R intervention compared to the Infant Massage (IM) intervention.

**Methods/design:**

This RCT will be multi-centre, paired and evaluator-blinded. Eligible subjects will be preterm or full-term infants with brain lesions, in first year of age with predefined specific gross motor abilities. Recruited infants will be randomized into CT-R and IM groups at baseline (T0). Based on allocation, infants will perform an 8-week programme of personalized CareToy activities or Infant Massage. The primary outcome measure will be the Infant Motor Profile. On the basis of power calculation, it will require a sample size of 42 infants. Moreover, Peabody Developmental Motor Scales-Second Edition, Teller Acuity Cards, standardized video-recordings of parent-infant interaction and wearable sensors (Actigraphs) will be included as secondary outcome measures. Finally, parents will fill out questionnaires (Bayley Social-Emotional, Parents Stress Index). All outcome measures will be carried out at the beginning (T0) and at end of 8-weeks intervention period, primary endpoint (T1). Primary outcome and some secondary outcomes will be carried out also after 2 months from T1 and at 18 months of age (T2 and T3, respectively). The Bayley Cognitive subscale will be used as additional assessment at T3.

**Discussion:**

This study protocol paper is the first study aimed to test CT-R system in infants at high-risk for CP. This paper will present the scientific background and trial methodology.

**Trial registration:**

NCT03211533 and NCT03234959 (www.clinicaltrials.gov).

## Background

Cerebral Palsy (CP), a clinical outcome linked to pre- or perinatal brain injury, represents the main chronic condition of disability in childhood [1, 2]. Disorders and disabilities associated with CP determine a relevant social, financial and emotional influence, on CP subjects, on their relatives, and also on health services, since these patients require continuous care, treatment and social support throughout their entire lives [3, 4]. The combined use of assessment tools such as the General Movement Assessment (GMA) according to Prechtl (GMs) and brain Magnetic Resonance Imaging (MRI) have shown high sensitivity and specificity in CP identification in infants since their first months of life [5]. In particular, a recent literature review has shown that brain MRI associated with GMA or neurological examination (Hammersmith Infant Neonatal Examination, HINE) performed at around full-term age determines the greatest predictive power of CP in high-risk newborns [6]. Early diagnosis has considerable importance because it allows for an early medical response and intervention which, as indicated by literature, can improve developmental outcome of high-risk children [7]. Moreover, early intervention (EI) is crucial because it targets brain plasticity, which for many functions has a maximum expressivity in an early limited time window or “critical period” [8, 9].

Several EI programs, based on Environmental Enrichment (EE) and Goal Directed approaches, have been used in clinical settings with positive results on neurodevelopment, but these findings are not conclusive due to high heterogeneity of clinical studies and applied intervention models [10–13].

In general, EIs should include certain essential characteristics to be effective and more specifically they should be early, intensive, personalized, multi-axial, family-centred and affordable for families and health services. In this context, biotechnologies and tele-rehabilitation appear to be promising approaches that can help achieve these standards [14].

Recently, in a European project (www.caretoy.eu, Trial Registration: NCT01990183), the CareToy (CT) system has been created. It is a technological and modular system able to provide, by means of tele-rehabilitation, a home and personalized intervention for very young infants (for further details see [15, 16]). CT allows subjects to carry out an early, intensive, individual EI carried out at home by parents consisting of highly customized exercises (rehabilitative packages called scenarios) remotely monitored by a clinical staff [15–18].

CT has been recently validated as a EI tool through a RCT study (CareToy training vs Standard Care), preceded by a pilot study, which involved a total of 61 in preterm infants born from 28 to 32 weeks of gestational age, without brain lesions and therefore considered at low risk for neurodevelopmental disorders. Children recruited in the pilot and RCT studies were divided into CT and Standard Care groups. Children allocated to the CareToy group followed a 4 weeks training with CT system, at the same time children allocated to the control group performed only Standard Care. All children were assessed with specific and standardized scales and questionnaires, immediately before and immediately after treatment period. Results of the study showed that CareToy training has a positive effect on promoting short-term visual and motor infant’s development [19, 20].

Based on these findings, the purpose of this study is to compare, through a Randomized Controlled Trial (RCT), the effects of two types of EI (CareToy training vs Infant Massage) on neurodevelopment of a group of at least 42 children at high risk for CP. The general hypothesis is that the CareToy system, with some adaptations, could be a useful EI tool also in brain-lesioned children at high risk for CP, effectively promoting motor, cognitive and perceptual development. In order to provide an EI to all children participating in the study, it was decided to offer Infant Massage (IM) as a valid alternative to CareToy. The choice of proposing IM was favoured by its ever-increasing application in Neonatal Intensive Care Units (NICU) [21]. The general hypothesis of IM intervention is that tactile stimulation (parent-infant) is able to promote neurodevelopment, emotional regulation of behavioural states and parent-infant relationship [22]. The rationale underlining these mechanisms seems attributable to increased metabolic efficiency and reduction of stress hormone synthesis. A recent systematic review [22] highlighted the effectiveness of IM on promoting neurodevelopment of preterm babies [23]; moreover, although studies on IM in infants with early brain damage are still limited, it seems to have positive effects on muscle tone and general motor development [24].

Based on this state of the art, the main purpose of this study is to evaluate the effects of CareToy EI (with a revised version of CareToy designed for this purpose), compared to those of Infant Massage, on neurodevelopment of infants at high risk for CP.

## Methods/design

This paper presents the protocol of an RCT which compares the effects of CareToy training to those of Infant Massage on neurodevelopment of infants at high risk for CP. Details of the two treatment protocols are described below.

### Ethical considerations

Tuscan Region Paediatric Ethics Committee (Italy) approved this study (no. 84/2017). Before the signing of informed consent, a dedicated personnel will verbally inform all parents of eligible infants about the trial, giving them also written informative material. Two informed consent forms during two different phases of the study will be provided. The first form allows for an observational phase related to a standardized GMA from the writhing period up to the fidgety one. Then, a second consensus form related to the intervention phase (CareToy or IM) will be given only to parents of infants with fidgety absent. There is a dual purpose for offering two treatments: to compare the two EIs and, for ethical reasons, to allow all eligible infants to carry out an EI.

### Primary objective

The main aim of the present trial is to explore the effects of CT training on neurodevelopment of infants at high risk of CP and then compare these effects to those of IM.

Three hypotheses have been specified:

1. CareToy Revised (CT-R) is a useful rehabilitative medical device for young infants (< 1 year) at high risk for CP
2. Infants receiving CT-R training will develop motor, perceptual and cognitive abilities faster than infants receiving IM
3. Improvement in visual and motor abilities will be faster during CT-R training than during IM.

The secondary aim is to investigate the different impact of CT-R on neuromotor development, both quantitatively (postural, motor and manipulation competences) and qualitatively (motor repertoire and adaptive abilities). Moreover, another purpose is to measure the efficacy of CT-R training on visual and cognitive development, parent-infant interaction and sleep-waking pattern.

The two phases of this project will be preceded by a small pilot study in which feasibility of EI effects of CareToy Revised system (CT-R training) compared with Infant Massage (IM) will be evaluated.

### Study design

To make a comparison between the effects of CT training and IM in brain-damaged infants, a multicentre, evaluator-blinded, paired RCT will be carried out.

The involved clinical centres are the Department of Developmental Neuroscience, IRCCS Fondazione Stella Maris in Pisa; the Neonatal Intensive Care Unit of University Hospital “Santa Chiara” in Pisa, the Neonatal Intensive Care Unit, Department of Perinatal Medicine of University Children’s Hospital “A. Meyer” and the Division of Neonatology, Careggi University Hospital, in Florence.

There will be two investigative arms: CT-R and IM training. Both training programs will last 8-week.

**Fig. 1:**
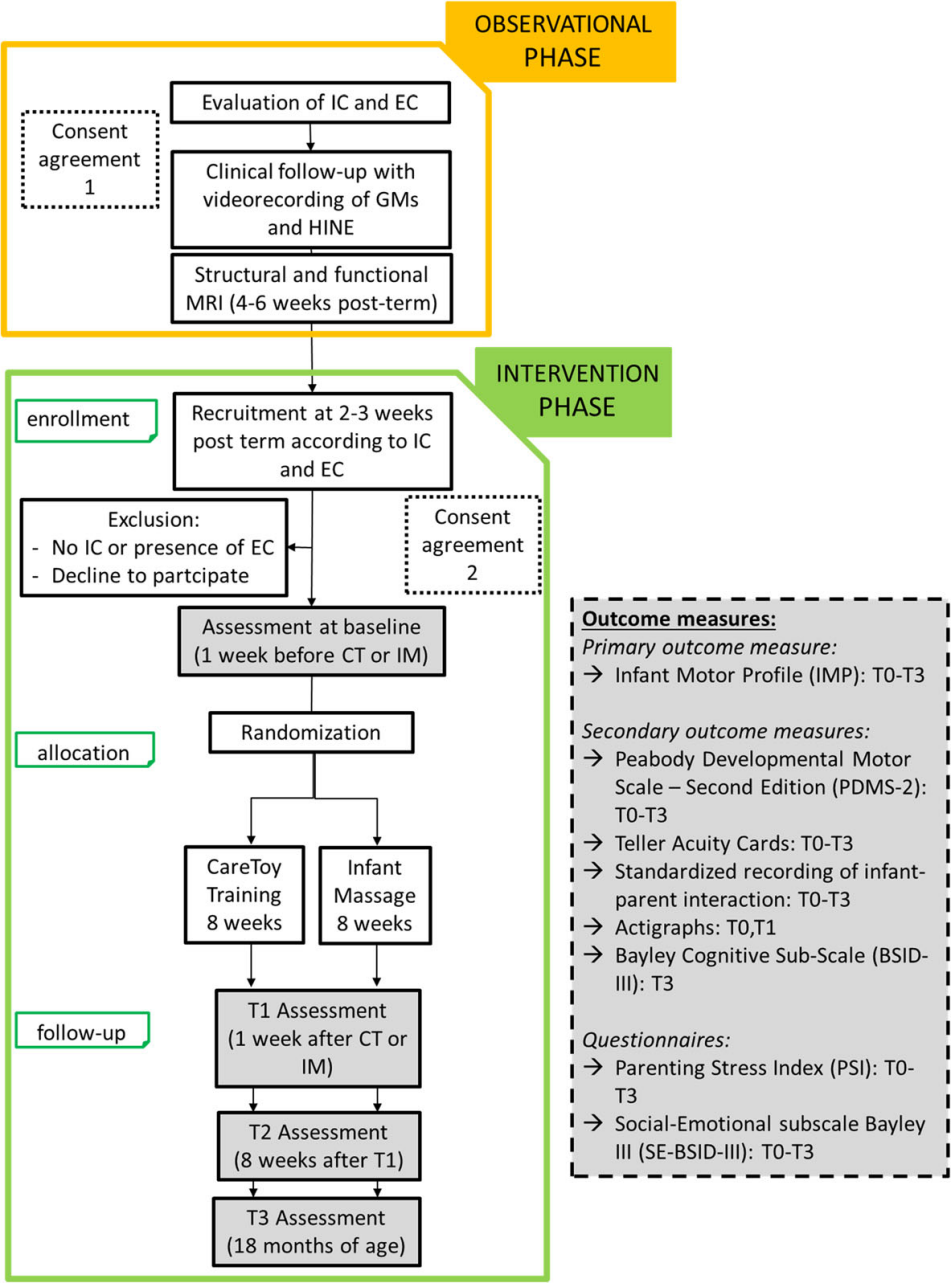
Study design

All the infants will be clinically assessed at T0 (baseline, in the week preceding CT-R training/IM) and at T1 (i.e. in the week after the end of the 8-week programme of CT-R training/IM). T1 will be the primary endpoint. Then, all infants will be followed at T2 (i.e. 8 weeks after the end of intervention period) and at T3 (i.e. at 18 months of age). Figure 1 shows the detailed timeline.

This study will be structured in two parts: an observational phase and an intervention one.

The observational phase will be aimed to early detect the brain-lesioned infants at high risk for CP through standardization of traditional clinical procedures, based on the most updated existing criteria. The following intervention phase will be aimed at verifying and comparing, with a RCT, the efficacy of two EI models (CareToy-Revised and Infant Massage) in promoting neurodevelopment of high-risk infants.

### Observational phase

The observational phase will consist of a standardized follow-up for monitoring development of infants through ultrasound evidence of brain injury verified at the two Neonatology Units involved in this project. Monitoring will also include neurological assessment (HINE) and recording of spontaneous activity according to Prechtl (GMA) [25, 26].

These examinations will be performed at around term period and also 2–3 months after post-term age, in the Neonatology Unit designated for regular clinical follow-up examinations.

Moreover, as clinical recommended for high-risk infants, a brain MRI during spontaneous sleep will be performed within 6 weeks post-term. MRI protocol foreseen by this project includes structural images, already present in clinical protocols adopted by the involved clinics and necessary for definitive inclusion in the second intervention phase, and a brief period of functional MRI acquisition [27–29]. It will include two further series of images (GRE-EPI, TR / TE = 3000/50, FA = 90 °, FOV = 240 × 240 mm, matrix = 96 × 96, thickness = 3 mm), for a total duration of 4 minutes, according to a block diagram, with an alternation of visual stimulus presentation suitable for assessing visual system integrity that is used in movement perception (MT area of the parietal cortex). More specifically, the passive stimulus consists of image presentations, through appropriate non-magnetic glasses, for MRI that simulates coherent or incoherent movement.

### Intervention phase

In the interventional phase, infants will proceed with an early intervention, which could be CareToy-R or Infant Massage, according to allocation group. Each intervention will last 8 weeks, 5 days a week; during this experimental phase all infants will continue to receive standard care foreseen by National Health System. All other specific interventions (such as physical therapy or other treatments) will be recorded in a specific diary. Therapy protocols are described below.

### Study sample and recruitment

Participants will be recruited at Tuscany Neonatology Units in Pisa (Santa Chiara University Hospital) and in Florence (Meyer and Careggi Hospitals). The neonatological staff will assess eligibility of infants on the basis of inclusion and exclusion criteria prior to hospital discharge and staff will inform parents about study. If a family expresses interested in participating in the study, they will receive an introductory letter and an informative flyer. Recruitment must take place within the first year of age of the infant and, because of the characteristics of the interventional setting and exercises, before the complete acquisition of the trunk control in sitting position. If parents are willing to participate, they will be asked to sign a first agreement for admission to the observation phase and another one to partake in the intervention phase. As indicated above, Tuscan Region Paediatric Ethics Committee approved the clinical trial. Moreover, CT-R, as the previous CT, has been classified as a medical device without an EC mark, so an approval of a new clinical trial was requested and obtained by the Italian Ministry of Health.

Inclusion criteria will be the following:

- Abnormal or specific neurological signs at neurological examination of GMA or HINE, associated with one of the following conditions

- Persistent brain ultrasound periventricular hyper-sonority
- Evidence of cerebral haemorrhage
- Evidence of Periventricular Leukomalacia (PVL)
- Cerebral stroke
- Moderate or severe asphyxia

Exclusion Criteria:

- Polymalformative syndrome or cerebral malformations
- Severe retinopathy (III-IV degree)
- Severe sensory disturbances (deafness or blindness)

Moreover, to be enrolled in the intervention phase, additional inclusion criteria will be:

- Persistence of abnormal Spontaneous GMs evaluation according to Prechtl at 2–3 months post-term (absent or abnormal FMs)
- Persistence of abnormal/specific signs according to HINE

Finally, the inclusion criterion for the start of intervention phase (CareToy-R or Infant Massage) is based on motor requirements defined on the basis of Ages & Stages Questionnaire scores. In general, skills required to start EI range from initial head control to complete trunk control in sitting position. In other words, infants who have no initial head control or have acquired the sitting position cannot be admitted to the intervention phase. In the first case, it is necessary to wait until the infant can control the head within the first year of age, in the second case they will not allocated to the intervention phase.

To monitor motor progress, families will receive the section on “gross motor skills evaluation” of Ages & Stages Questionnaire (ASQ-3; gross motor [30]); as soon as the child reaches some pre-established motor skills (see below). Treatment can then start according to assignment group.

- score ≥ 10 from 3 to 4 months
- score ≥ 5 < 50 from 5 to 6 months
- score ≥ 10 < 30 from 7 to 8 months

The exclusion criterion is represented by a worsening of general clinical situation for intercurrent medical conditions or an onset of epileptic seizures.

### Sample size

Based on the chosen primary outcome measure, Infant Motor Profile (IMP [31],), and on IMP results of a pilot study and of previously conducted RCT in preterm infants without congenital brain lesions [15, 19, 20] and considering a 80% power and a significant level of 0.05 and a effect size of at least 0.6, a sample of 38 children is required. However, considering also a 10% of drop-out rate, a minimum of 42 infants will be required. In order to facilitate parental work and participation in the project, eligible twins will be allocated into the same group.

### Randomisation

Randomization will be done after enrolment: participants will be randomly assigned to CT-R training or IM group by the use of an automatic generation of 1:1 sets. A third party blind to clinical aspects of trial will manage these sets, sealing them in numbered envelopes.

### Blinding

Parents, therapists and clinical staff will be aware about the allocation group of infants. Assessors who will evaluate infants with outcome measures (IMP; Peabody Developmental Motor Scales - Second Edition (PDMS-2); Bayley Scales of Infant Development III Edition, BSID-III) will use the video-recording of the assessment session so they will be absolutely blind.

### Therapy protocols

#### CareToy-R training

As in a previous study [20], the clinical staff, mainly composed by child neurologists and paediatric physical therapist will organize customized goal-directed rehabilitative activities (i.e. CareToy scenarios) to be done in three different possible position which could be sitting, supine or prone, mainly focused on: postural abilities (e.g. postural control, rolling...), manipulation capabilities (e.g. reaching, grasping, manipulation…) and visual functions (e.g. visual attention and orientation…).

The new CT system, i.e. the revised version (CT-R), has been planned and designed in order to adapt the CareToy system to this new population by integrating a support for posture maintenance. Based on postural needs of brain-lesioned infants, a modular system called Siedo & Gioco (Fumagalli, Italy) has been incorporated. Siedo & Gioco system is a soft multifunctional system which allows the infant or a small child to be placed in many different confortable and safe positions (e.g. supine, prone, sitting or on one side). The Siedo & Gioco system is composed a set of soft and coloured modules with different shapes which can be attached to a mat by Velcro straps placed on modules and on mat. Technical specification for optimize suitability of Siedo & Gioco to CT system have been provided and an ad-hoc set of modules has been created in order to offer postural and perceptive stimuli. Thanks to the flexibility of this integrated system, the modularity of CT platform in this revised version (CT-R) has been maintained.

According to the initially established activities and modules (the activities and consequently the modules will be periodically updated), the CT-R system will be customized and delivered to the family’s home. CT-R training will be structured in 8 weeks, in which there will be about 30–45 min of daily planned activities, consisting of different scenarios lasting 2–10 min each.

Parents will be instructed to carry out the daily training during the wake and active periods of their infants. It will be remotely supervised by the clinical staff, who will have the possibility to remotely manage the CT scenarios based on each infant rehabilitation requirement and improvement during the 8-weeks training. Each scenario will be scored at the end by the parents on the basis of a questionnaire recording the infant’s acceptance and compliance.

Rehabilitation staff will train parents to use the system and to interact with their infant during the first 5 days of training and once a week in the following weeks.

#### Infant massage

Infant massage consists generally of a systematic touching by hands on different parts of the body and is characterized by slowness and gentleness [22]. IM is often associated to other forms of “contact” with the infant, e.g. kinaesthetic (arm and leg passive extension/flexion), auditory, verbal or visual stimuli.

Families assigned to IM group will perform a training course of 4–5 sessions lasting about one hour each. The massage will be done on different body areas and illustrated material on how to perform massage sessions at home will be provided to families. Parents will be required to perform IM 5 days a week, depending on the child’s willingness, for 8 weeks, and to record massage frequency in a dedicated diary.

### Outcome measures

As previously reported, infants will be assessed according to the timeline (Fig. 1). The primary outcome measure, assessing the motor development, will be the IMP. The selected secondary outcomes will be the: PDMS-2, BSID-III Cognitive subscale, standardized video-recordings of parent-infant interaction, Teller Acuity Cards® and Actigraphic analysis (Motionlogger Microwatch). Due to multiple outcomes and variable willingness of infants, each evaluation time would be completed, if necessary, in two successive days to guarantee greater compliance.

### Primary outcome measure

#### Infant motor profile (IMP)

IMP is a video-recorded motor evaluation of the infant placed in different positions (supine, prone, sitting, standing while grasping and manipulating objects). It is composed at first by observation of spontaneous activity and then by stimulation of motor abilities and manipulation of objects in different position. It consists of 80 items, subdivided in five motor domains, which allow for a calculation of a total IMP score. IMP does not only evaluate motor performance in quantitative terms, but also provides qualitative assessments, such as movement variability and fluency and adaptability of motor strategies.

It is suitable for infant born at term and preterm from 3 months of age to until the child has acquired good autonomous skills (which is approximately 18 months).

Previous studies with CT have had the IMP as the primary outcome measure. It will be carried out at all assessments as shown in Fig. 1.

### Secondary outcome measures

According to secondary goals, the following measures have been selected.

### Motor assessment

#### Peabody developmental motor scales - second edition (PDMS-2)

It evaluates fine and gross motor movements from birth to 5 years. The scale is made up of 6 subtests (reflex, sitting, walking, manipulation of objects, grasping and visual-motor integration), whose results are combined into 3 global motor performance quotients: Gross Motor, Fine Motor and Global Motor [32]. It will be performed at all time-points (T0, T1, T2 and T3).

### Cognitive assessment

#### BSID-III-cognitive subscale

It is a standardized scale for the cognitive development assessment in 1–42 months old children [33]. It will be performed at T0 and T3.

### Visual function assessment

#### Teller acuity cards®

It is a test widely used in young infants and non-collaborative subjects to estimate visual acuity. The trained assessor proposes a series of black and white stripes cards with different widths on one side and neutral stimulus (grey background) on the other side to the infants, judging his/her visual attention. It evaluates the skill to look at a visual target. The estimated visual acuity is the thinnest width of stripes that the child is able to fix and prefer to the neutral grey area. The test is based on the “preferential looking” concept; response indicators are on a spontaneous behaviour basis, i.e. head positioning and eye directing to stimulus. The test is highly reliable, versatile, and it can be executed in a few minutes [34, 35]. It has been used in scientific studies for evaluating the results of training on visual development [36]. It will be performed at T0, T1, T2 and T3. A further complementary visual assessment will be carried out by means of an eye-tracker (CareToy C) [37] that allows a measurement of fixation and pursuit.

### Parent-infant relationship assessment

#### Standardized video-recordings of parent-infant interaction

A video of a free play interaction of about 3–5 min between parent and child will be carried out. Videos will be classified by expert certified raters according to Child – Adult Relationship Experimental Index (CARE – Index) [38] and/or Emotional Availability (EA) Scales [39], blinded to allocation group. They will be carried out at T0, T1, T2 and T3.

### Study of organization and maturity of sleeping

#### Movement recording with Actigraphs

Actigraphs is the simplest and least invasive measurement tool to evaluate sleep which allows for protracted monitoring (from days to months) [40]. It uses accelerometric sensors similar to a wristwatch and it is generally worn on the non-dominant side (hand or wrist). Motionlogger Microwatch will be used and combined with Sadeh algorithm for sleep evaluation. Clinical data such as total sleep time (TST), waking after sleep onset (WASO), and sleep efficiency (SE) will be calculated during the training. It will be worn on an infant’s ankle for 1 week at T0 and T1.

### Questionnaires

#### BSID-III social-emotional scale

This is a subscale of the Bayley III. It is a screening tool for early identification of social-emotional deficits in subjects from birth to 42 months. It is a questionnaire to be completed by parents and it allows an evaluation of child emotional-functionality, communicative needs, interactive relationship and use of interactive emotions to social problem-solving [41]. This scale will be done by the parents at T0, T1, T2 and T3.

#### Parenting stress index (PSI)

This questionnaire, containing 36 items, is divided into 3 subscales and requires about 5–10 min to fill out. It detects defined features of parents, of their children and of their environment that are frequently related to parenting stress. It is a tool, widely used for early detection of parent-child dysfunction in their relationships [42]. It will be filled in by the parents at T0, T1, T2 and T3.

### Analyses

Statistical Package for Social Sciences (SPSS) will be adopted for carrying out the statistical analyses. Descriptive statistics will be performed to create the record of the data for each group. Potential baseline differences and between group differences will be explored computing the *p* values. When necessary, Bonferroni correction will be applied. Firstly, delta changes immediately after treatment (T1, primary endpoint) vs baseline will be calculated to assess the short-term effects of CT-R training versus IM in primary and secondary outcome measures, taking account as covariates base level of motor development, type and grading of brain lesion, family compliance and dose of intervention. After, multivariate statistics will be performed.

## Discussion

This paper shows rationale and protocol for an RCT aimed at evaluating the efficacy of a new EI tool, called CT-R, respect to IM in brain-damaged infants.

It is built on several studies carried out over the last years [15, 17–20, 43] in infants without brain lesions. According to our experience, there are some limitations in performing high-quality home-based EI that could be different from family to family because home environments are various and difficult to control. On the other hand, the advantages for families are that they do not have to go to a clinic for every intervention session and they can personally learn how to stimulate their infant in their own home. In our proposal, the abovementioned disadvantages can be overcome thanks to the option of remotely managing home-training from the clinic, in this way parent behaviour can be controlled and EI will have greater access.

CT-R system is a completely new technological tool for delivering at home personalized EI in infants. The precise study protocol, that follows the CONSORT guideline [44, 45] and the comparison with another EI, will permit to scientifically assess the effects of CT-R intervention on neurodevelopment in a sample of brain-lesioned infants.

## Abbreviations

ASQ-3: Ages & Stages Questionnaire – 3
BSID-III: Bayley Scales of Infant Development – III
CARE Index: Child – Adult Relationship Experimental Index
CP: Cerebral Palsy
CT: CareToy
CT-R: CareToy Revised
EE: Environmental Enrichment
EI: Early Intervention
FMs: Fidgety Movements
GMA: General Movement Assessment
GMs: General Movements
HINE: Hammersmith Infant Neonatal Examination
IM: Infant Massage
IMP: Infant Motor Profile
MRI: Magnetic Resonance Imaging
NICU: Neonatal Intensive Care Units
PDMS-2: Peabody Developmental Motor Scales - Second Edition
PSI: Parenting Stress Index
RCT: Randomised Controlled Trial
